# 

**Published:** 1874-03

**Authors:** 


### CONTENTS:

ORIGINAL COMMUNICATIONS:

ARTICLE I.—Infants. Their Food and its Digestion. By Alex. S. von Mansfelde, M.D., Jefferson, Ill........... 129

ART. II.—Postural Treatment for Extensive Distension of the Intestines with Gas. By C. T. Parkes, M.D... 143

ART. III.—Labor, Puerperal Fever, and Death. By Geo. J. Monroe, M.D... 145

ART. IV.—Case of Dislocation of the Ulna backward, etc. By N. Bridge, M.D............ ..................... 147

PROGRESS IN MEDICAL SCIENCES:

ART. I.—Report on Gynæcology. By A. Reeves Jackson, M.D........... 149

ART. II.—Progress of Surgery. By J. E. Owens, M.D.................... 153

SELECTIONS:

Parasitic Origin of Disease.... ....... 161

REPORTS OF SOCIETIES:

Chicago Society of Physicians and Surgeons.............................. 173

EDITORS' BOOK TABLE............... .. 176

EDITORIAL................................ 182

Historical Sketch of Rush Medical College; from Address of Pres. Freer to Graduating Class, Feb. 17, 1874.... 187

Mortality Statistics ............. ....... 189

